# Transcriptome-Wide Analyses Identify Dominant as the Predominantly Non-Conservative Alternative Splicing Inheritance Patterns in F1 Chickens

**DOI:** 10.3389/fgene.2021.774240

**Published:** 2021-12-03

**Authors:** Xin Qi, Hongchang Gu, Lujiang Qu

**Affiliations:** Department of Animal Genetics and Breeding, National Engineering Laboratory for Animal Breeding, College of Animal Science and Technology, China Agricultural University, Beijing, China

**Keywords:** chicken, hybridization, RNA-seq, dominant, alternative splicing, inheritance pattern

## Abstract

Transcriptome analysis has been used to investigate many economically traits in chickens; however, alternative splicing still lacks a systematic method of study that is able to promote proteome diversity, and fine-tune expression dynamics. Hybridization has been widely utilized in chicken breeding due to the resulting heterosis, but the dynamic changes in alternative splicing during this process are significant yet unclear. In this study, we performed a reciprocal crossing experiment involving the White Leghorn and Cornish Game chicken breeds which exhibit major differences in body size and reproductive traits, and conducted RNA sequencing of the brain, muscle, and liver tissues to identify the inheritance patterns. A total of 40 515 and 42 612 events were respectively detected in the brain and muscle tissues, with 39 843 observed in the liver; 2807, 4242, and 4538 events significantly different between two breeds were identified in the brain, muscle, and liver tissues, respectively. The hierarchical cluster of tissues from different tissues from all crosses, based on the alternative splicing profiles, suggests high tissue and strain specificity. Furthermore, a comparison between parental strains and hybrid crosses indicated that over one third of alternative splicing genes showed conserved patterns in all three tissues, while the second prevalent pattern was non-additive, which included both dominant and transgressive patterns; this meant that the dominant pattern plays a more important role than suppression. Our study provides an overview of the inheritance patterns of alternative splicing in layer and broiler chickens, to better understand post-transcriptional regulation during hybridization.

## Introduction

Splicing of pre-mRNA is a crucial post-transcriptional process that increases proteome diversity in eukaryotes. Alternative splicing (AS) generates multiple isoforms from a single gene using different combinations of exons. AS is a widespread and complex component of gene regulation in humans and domestic animals, and increasing evidence suggests that aberrant AS functionality can be the cause or consequence of many diseases, and may also associating with economically important traits in domestic animals (Pan et al., 2008; Merkin et al., 2012; Gao et al., 2018; Dlamini et al., 2021). Therefore, splice-altering therapies using animal models have been extensively studied for many diseases such as neurodegeneration and muscular dystrophies, and AS events have also emerged as new biomarkers in some circumstances (Montes et al., 2019; Zhao, 2019). Changes in AS are regulated by the interactions between cis- and trans-acting elements, and studies in *Camellia* and *Drosophila* suggest that parental genetic divergence may affect the regulation patterns in hybrids due to these interactions (Coolon et al., 2014; Zhang et al., 2019). Therefore, it is important to study the AS regulatory mechanisms in birds.

Based on different combinations of the constitutive and alternative exons, AS is divided into seven types: exon skipping (SE), intron retention (RI), alternative 5′ splice sites (A5SS), alternative 3′ splice sites (A3SS), mutually exclusive exons (MXE), alternative promoters (AFEs and ALEs), and alternative polyadenylation (tandem 3′UTRs). SE is the most prevalent AS event in approximately 40% of higher eukaryotes, and commonly generates functional isoforms, while RI is the dominant type in plants (Barbazuk et al., 2008; Weatheritt et al., 2016; Cardoso et al., 2018; Chen S.-Y. et al., 2019). With the rapid development of sequencing technology, a broad range of bioinformatics approaches can identify and classify AS events using isoform-based and count-based methods, of which several tools perform robustly and exhibit excellent overall performance (Mehmood et al., 2019). However, there is a lack of systematic analysis of AS in chickens, and it is necessary to study the components and divergence patterns of splicing events, providing an alternate view of transcriptome plasticity.

Hybridization is ubiquitous in nature―involving more than 25 and 10% of plants and animals, respectively―and widely utilized in breeding programs (Whitney et al., 2010). Some hybrids show enhanced environmental adaption and growth rate, whereas others are infertile or exhibit negative economic traits (Abasht and Lamont, 2007; Chen, 2010; Chen et al., 2013; Zhang et al., 2015; Clasen et al., 2017). Hybridizing two different strains can remodel the parental gene patterns, with the genes in hybrids diverging from the mid-parental value, leading to “transcriptome shock” (Hegarty et al., 2006; Han et al., 2014; Cui et al., 2015). These genes mainly contribute to some transgressive phenotypes called over- and under-dominant genetic patterns (Zhuang and Tripp, 2017). Additive and dominant patterns also represent phenotypical variations, while conserved patterns show parental similarity. To take advantage of genome-wide methods for expression analysis, the classic hypotheses of inheritance, dominance, over-dominance, and epistasis should have more contributions at the molecular level to explore the mechanism of heredity (Shull, 1908; Bateson, 1910; Jones, 1917; Mcmanus et al., 2010). However, the identified predominant genetic patterns regulating phenotype divergence are not always consistent among studies because of different genetic backgrounds, species, and tissues employed in those studies. Studies on *Camellia* and coffee have indicated that the non-additive expression prevailed over other patterns (Marie-Christine et al., 2015; Zhang et al., 2019). On the other hand, several studies have suggested that additivity is the predominant genetic pattern in maize, rice, and cotton (Swanson-Wagner et al., 2006; Li et al., 2008; Rapp et al., 2009), while divergent outcomes suggest that additive or high parent-dominance is the major pattern in chickens (Mai et al., 2019; Zhuo et al., 2019). Most previous studies have identified different inheritance patterns based on gene expression, and there is rarely a transcriptomic study that investigates AS event patterns, considering the association between AS and gene regulation at the post-transcriptional level.

In this study, Cornish Game (CG) and White Leghorn (WL), representing broilers and layers, respectively, were used as the parental strains to produce purebred and reciprocal crossed progenies. Taking advantage of RNA-sequencing (RNA-seq), tissue samples from the brain, liver, and breast muscle were collected and sequenced. Splicing events from each sample were identified, classified, and quantified, and events significantly different between purebreds were detected for further study. Finally, five main types of inheritance patterns―conserved, additive, parental-enhancing/suppressing, dominant, and transgressive―were categorized, and compared between purebred strains and hybrid crosses. Changes in alternatively spliced genes indicated that the diverse AS inheritance patterns have different influences on heredity, and variation during hybridization. AS is an effective and novel approach for investigating genetic patterns, and understanding the molecular mechanisms of heterosis.

## Materials and Methods

### Sample Collection and RNA Sequencing

We used CG, a broiler breed with superior growth and muscle development, and WL, a layer breed with high egg production, acquired from the National Engineering Laboratory for Animal Breeding of the China Agricultural University. The four breeding patterns used in this study resulted in pure-bred progeny, CG, and WL, representing the first generation (F1) with parents of the same type, and reciprocal cross progeny WL ♂ × CG ♀ (LC), and CG ♂ × WL ♀ (CL) representing F1 hybrids (Supplementary Figure S1). Each group had six offspring (three female and three male), except CL where only two females were obtained. We collected the brain, breast muscle, and liver from 23 one-day-old chicks, and extracted total RNA from the tissue samples using Trizol reagent (Invitrogen, Carlsbad, CA, United States). The DNA and RNA quality was assessed using a NanoDrop 2000 spectrophotometer (Thermo Fisher Scientific Inc., United States) and agarose gel electrophoresis. After synthesizing cDNA, PCR amplifications, and library construction, total RNA was sequenced, using paired-end 100-bp reads with a 300-bp insert, on an Illumina HiSeq 2500 platform (Illumina Inc., San Diego, CA, United States) using standard Illumina RNA-seq protocols.

### RNA-Sequencing Data Alignment and Alternative Splicing Analysis

The RNA-seq data were aligned to the chicken reference genome (Gallus_gallus-6.0) using STAR v2.7.5 (Dobin et al., 2012). Duplicate reads were removed to eliminate potential bias, using SAMtools (Li, 2009; Dozmorov et al., 2015). Putative AS events were detected and annotated from aligned RNA-seq data using rMATS v4.1.0 (Wang et al., 2017). Five major types of AS events were identified: A3SS, A5SS, RI, MXE, and SE. To quantify and compare event variation, the percent spliced-in (PSI) value of each AS was calculated for each sample using reads on target and junction counts, where PSI was equal to, the number of reads specific to exon inclusion isoform divided by the sum of reads specific to exon inclusion and exclusion isoforms. Moreover, a hierarchical model for paired replicates and false discovery rate (FDR) correction (FDR < 0.05) was used to determine the statistical differences between the parental strains (Shen et al., 2012). Only the events occurring on autosomes were considered in this study because of incomplete dosage compensation in the chicken sex chromosome. Additionally, if the sum of inclusion and skipping read counts is less than 10 (average in replicate samples), AS was considered low quality and then filtered. Liver samples from the hybrid females were removed because the detection process for putative AS events provided abnormal results.

### Classification of Alternative Splicing Inheritance Patterns

To measure differences between parents and hybrids in order to identify inheritance patterns of AS, samples were recombined as male cross (MC: CG ♂, WL ♂, CL ♂), female cross (FC: CG ♀, WL♀, CL♀), male reciprocal cross (MR: CG ♂, WL ♂, LC ♂), and female reciprocal cross (FR: CG♀, WL♀, LC♀). First, shared AS was determined by merging expressed events in hybrids with significantly different (FDR < 0.05) events in pure-bred, and calculating the average PSI value for biological replicates. A 1.25-fold threshold was set as the criterion for classification as conserved or non-conserved splicing (Gu et al., 2020). Non-conserved AS was classified into eight types: events for which quantification in the hybrid was less than in CG and greater than in WL (or vice versa) was defined as additive (2 types); splicing for which quantification in the hybrid was remarkably higher or lower than in one of the parents and similar to that in another was categorized as dominant (4 types); and splicing for which quantification in the hybrid was significantly greater or lesser than in both parents was classified as transgressive inheritance (two types). Non-conserved genes which were identified in the same mode in more than two groups in a tissue, would be considered as exhibiting that inheritance mode in the corresponding tissue. All eight AS types are listed in Supplementary Figure S1. Exploring the biological function of these genes further, Gene Ontology (GO) classification and enrichment analysis were performed using PANTHER v14 (Thomas et al., 2003), and Kyoto Encyclopedia of Genes and Genomes (KEGG) pathway analysis was carried out using the KOBAS v3.0 (Bu et al., 2021).

### Statistical Test of Inheritance Patterns

R (v4.0.2) was used for most of the statistical analyses in this study. Principal component analysis was performed using Prcomp for statistically different AS between the parental lines. Besides, the Venn diagram was visualized by the “VennDiagram” package, and heat-map was prepared using “pheatmap,” with hierarchical clustering among samples performed by “hclust.” After classifying inheritance patterns, the Kruskal–Wallis test was carried out to identify differences among the three tissues, while the Mann–Whitney test was used to compare divergence between CG/WL-like dominant, up/down-regulation dominant, and over/under-dominant cases in each tissue.

## Results

### Alternative Splicing Divergence Between Cornish Game and White Leghorn

We collected RNA-seq data from brain, liver, and breast muscle tissues of two inbred chicken strains, CG, and WL, representing parental lines, as well as their reciprocal crossed progenies. There was 246.3 Gb of RNA-seq data and 3.6 million mapped reads for each sample. After filtering low-quality reads using the NGS QC Toolkit v2.3 (Patel et al., 2012) and mapping them onto the reference genome, we obtained, on average, 22.8, 21.3, and 17.8 million mapped reads per individual for the brain, muscle, and liver tissues, respectively. AS events were quantified as PSI and classified into five types―A3SS, A5SS, MXE, RI, and SE―while statistically significant differences between WL and CG were identified.

**FIGURE 1:**
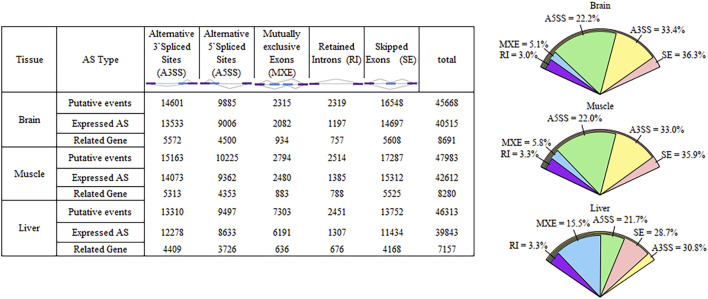
Overview of alternative splicing in Cornish and White Leghorn.

**FIGURE 2:**
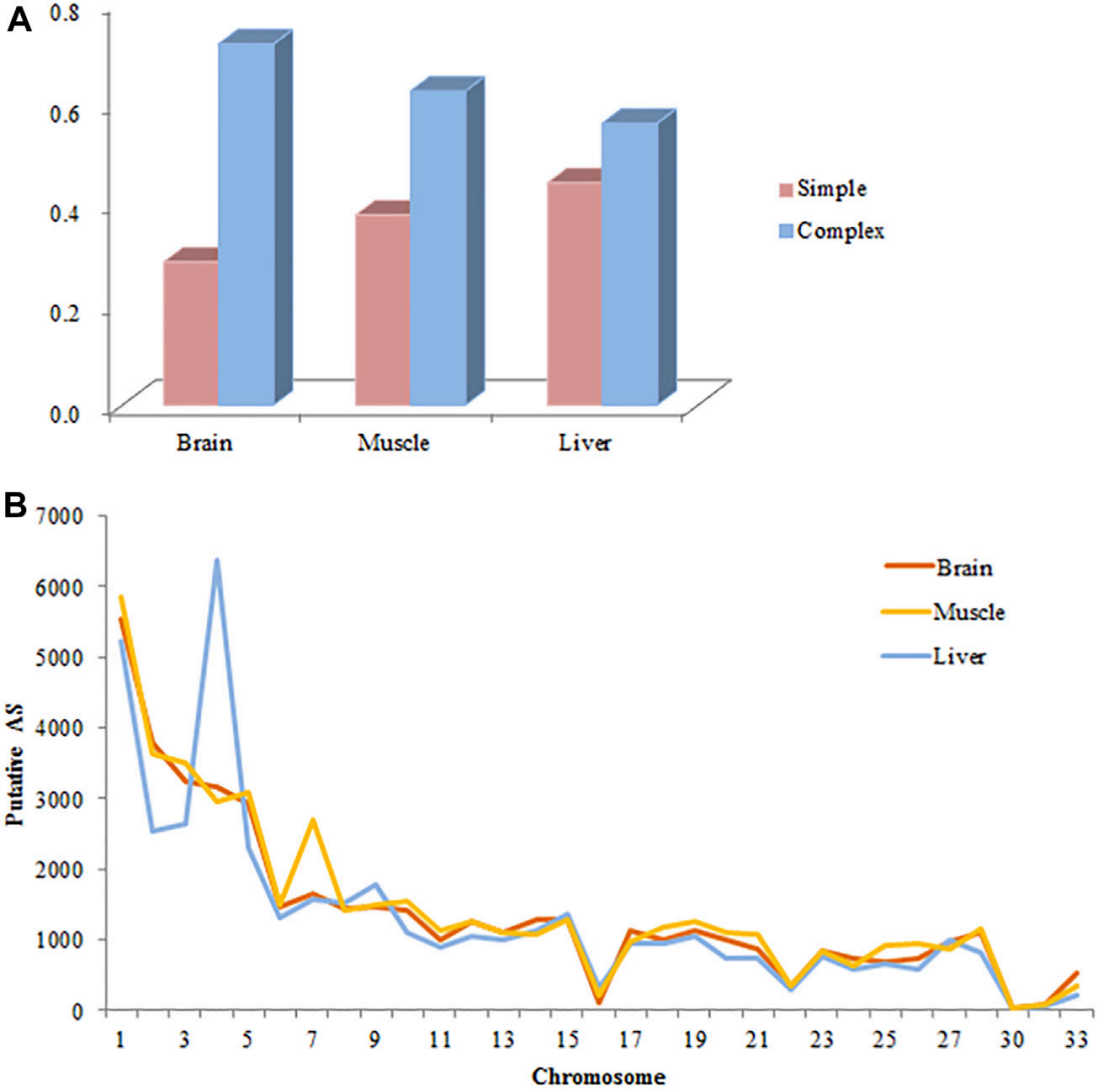
Basic information of alternative splicing events in each tissue. **(A)** Complexity of AS events per gene. **(B)** The distribution of AS events in the chicken genome. The distribution of 45668, 47983, and 46313 putative AS events identified from brain, muscle and liver on the chicken autosomes are shown. Most of the events distributed primarily on chromosomes 1 and 2.

**FIGURE 3:**
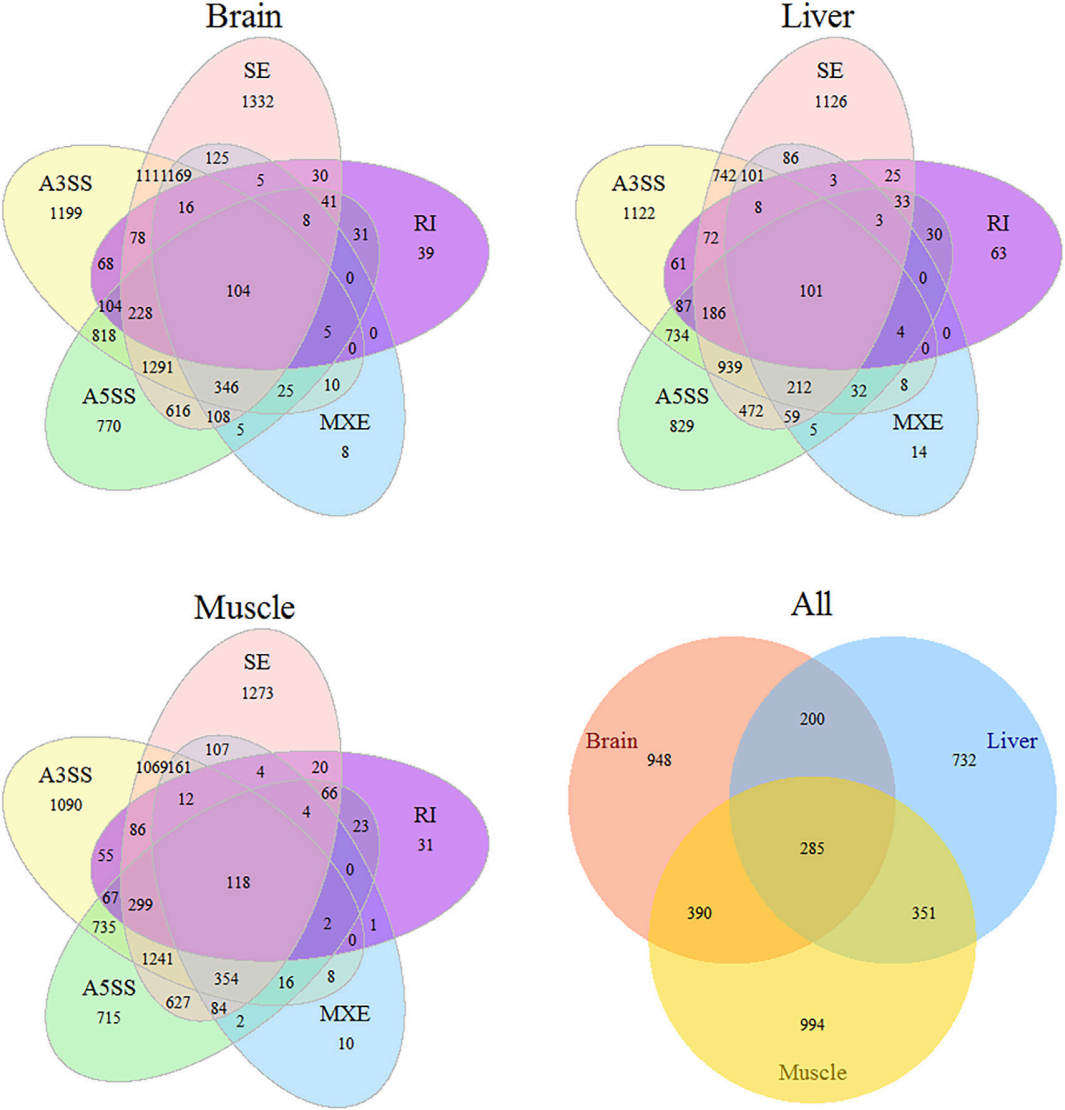
Venn diagrams illustrating statistical results of different types AS in tissue and divergence AS among tissues.

The number of putative splicing events was 45668, 47983, and 46313 in the brain, muscle, and liver tissues, respectively, most of which were expressed (86%), and related to more than 7000 genes (Figure 1). SE formed a large proportion of splicing in the brain and muscle tissues (approximately 36%), A3SS was slightly higher than SE in the liver tissue, and RI only accounted for 3% of the tissues. 28, 38, and 44% of genes only underwent one splicing (simple event), and over 56% of events among the tissues were complex events (Figure 2A). Most of the events were primarily distributed on chromosome-1 and numbered over 5200, while those located on chromosome-30 were less than 50 in number. In addition, over 6000 event locations were positioned on chromosome-4 in the liver and over 2700 in chromosome-7 in muscle tissues (Figure 2B). On the other hand, several genes participated in more than two types of events; 100 genes covered five types, with approximately 60% related to multiple types of events, among the three tissues (Figure 3). To summarize, locations of alternatively spliced events widely exist in tissues, and have a complex correlation with genes.

**FIGURE 4:**
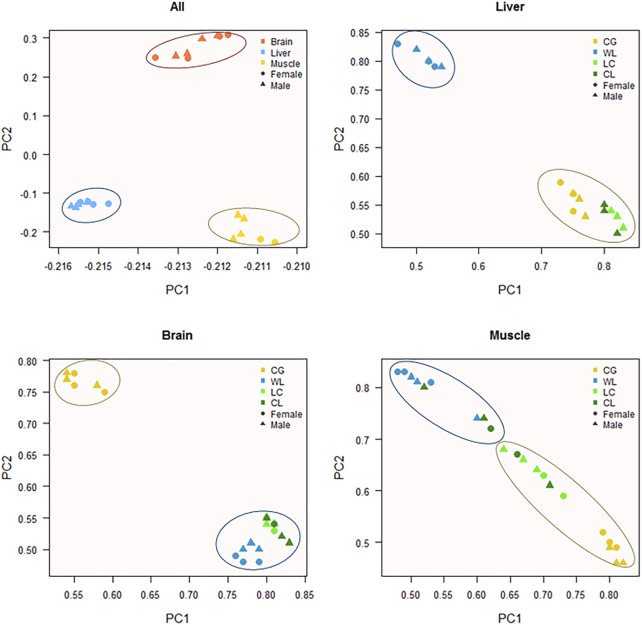
Principal Component Analysis of alternative splicing. PCA analysis is performed using PSI value for significantly different (FDR < 0.05) AS events between purebreds, which events located on the sex chromosomes has been excluded.

**FIGURE 5:**
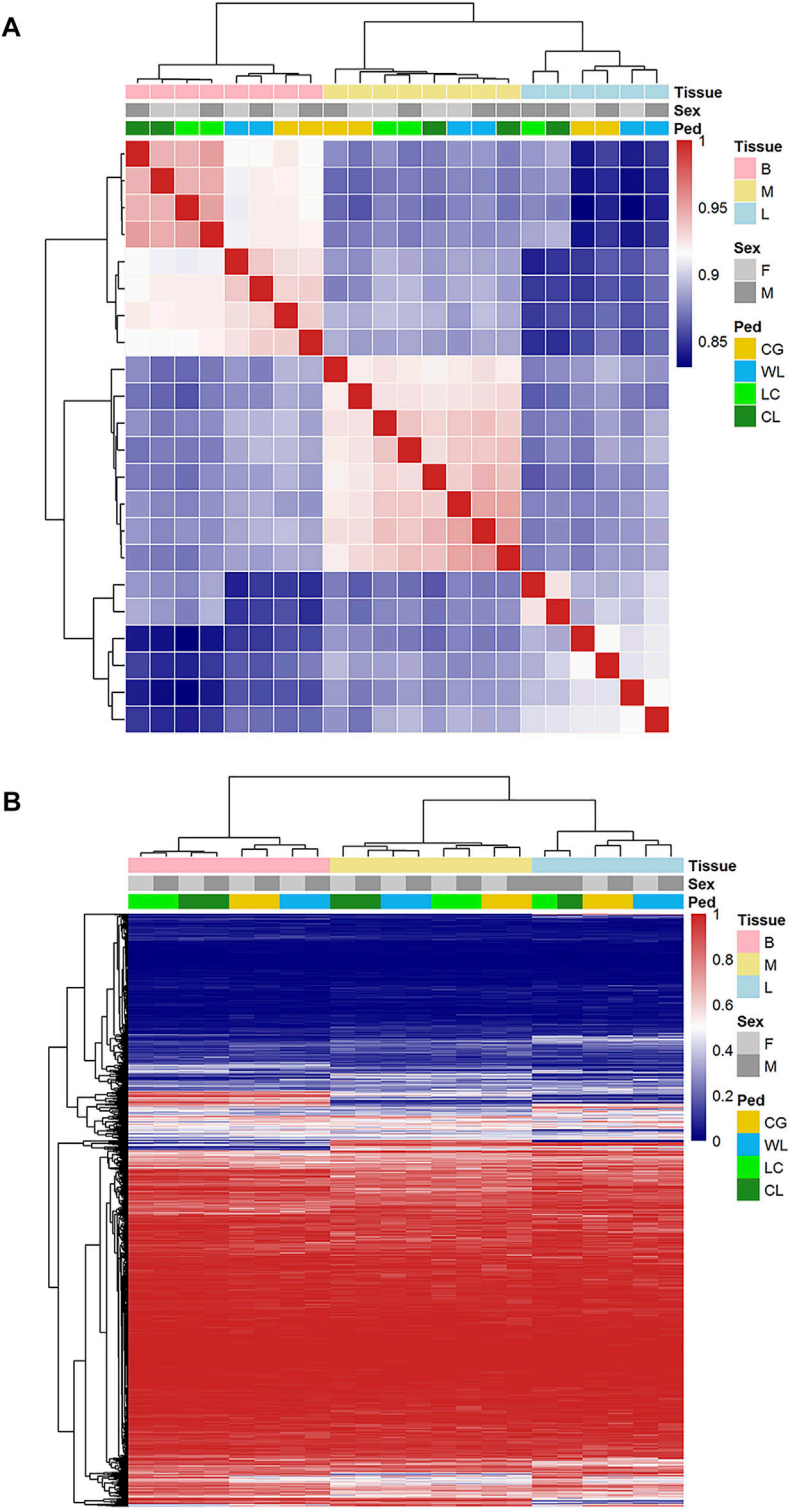
Correlations and hierarchical clustering of alternative splicing. **(A)** Spearman correlation based on alternative splicing (PSI) expressed in brain, liver, and muscle. **(B)** Hierarchical cluster for AS events in each group.

From among 40000 expressed AS, we used a hierarchical model to detect 2807, 4242, and 4538 (FDR < 0.05) events as significantly different between the two strains, in the brain, muscle, and liver tissues, respectively. These spliced events related to 1823, 2020, and 1568 genes, approximately half of which could be tissue-specific. In addition, the number of up-regulated splicing events was 3.4–5.5% (1387, 2038, and 2188), while down-regulated events accounted for 3.5–5.9% (1420, 2204, and 2350), between layers and broilers. Based on PSI values of divergence events in each sample, principal component analysis was performed (Figure 4); the tissue was significantly influenced by AS, and the strain probably played an important role in the liver and brain. The parent-of-origin effect might have influenced muscle formation because hybrids were observed to be slightly closer to the maternal group, while it showed insignificant influence on sex determination of the offspring. Further, hierarchical clustering using Spearman correlation―where the samples were classified into three tissue-based clusters and purebred were always categorized in the same group―showed that tissue- and strain-based clustering tended to be stronger than sex-based clustering (Figure 5).

### Classification of Alternative Splicing Inheritance Patterns

**FIGURE 6:**
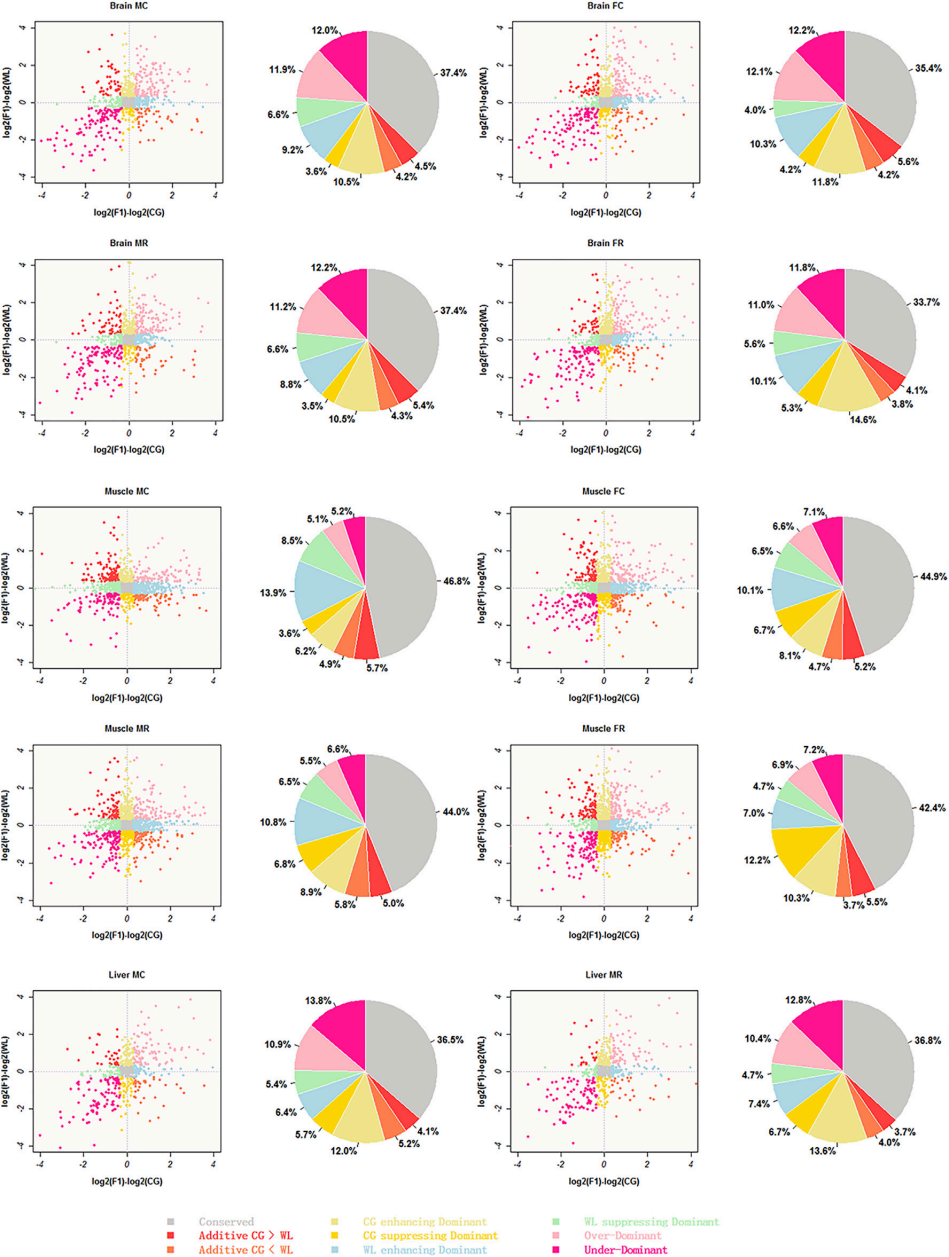
Scatter-plot and pie representing different inheritance patterns. inheritance patterns identified from reciprocal crosses and parental lines brain, muscle, and liver tissues were classified into nine clusters listed at the bottom of the images, respectively. Scatterplots represent relationships between F1 hybrids and their two parents, and pie charts show the relative proportions of these patterns for each cluster using different colors.

After filtering and removing sex chromosome splicing, AS between parental strains was merged with expressed splicing in hybrids; 754 splicing events in the liver were used for further analysis as against 1030 and 1950 AS events in the brain and muscle because only liver samples from male hybrids were available. Based on the difference in expression between parental and hybrid splicing, events were categorized into nine types in all four groups (MC, MR, FC, and FR) (Figure 6).

Based on statistical results (Table 1), we detected a significant difference in the sum of conserved, additive, WL-like dominant, and enhancing/suppressing dominant patterns among the three tissues (Kruskal–Wallis test, *p*-value ≈ 0.02 < 0.05), whereas there was no divergence in the number of CG-like dominant (*p*-value = 0.51) and transgressive patterns (*p* = 0.11). Conserved splicing was predominant, with above 34% in all three tissue types, while additive splicing accounted for a small proportion of non-conserved patterns, with an average of 8.7, 9.3, and 8.0% in the brain, muscle, and liver tissues, respectively. A greater proportion of events between the hybrids and their parents exhibited a non-additively expressed pattern, in which the sum of parental dominance was approximately 30%, and transgressivity accounted for approximately 22.7, 11.6, and 22.5% in the brain, muscle, and liver tissues, respectively. Therefore, up-regulated dominance was higher than down-regulated, in muscle and brain tissues (Mann–Whitney test, *p*-value = 0.03 < 0.05). Interestingly, the relative proportion of CG-like dominance was larger in brain tissues of the female groups and liver tissues of the male groups, while WL-like dominance was greater in the brain and muscle tissues of the male groups. No differences were detected between over-dominance and under-dominance in each tissue type. To check whether our conclusion was sensitive to specific statistical methods, we used Fisher’s exact test to identify events with significant divergence between hybrids and parents (Supplementary Table S1), with the resulting *p*-values controlled for an FDR (FDR < 0.05). Overall, we drew the same conclusion that most non-conserved events were dominant, with transgressive modes during hybridization, while a few events displayed additive patterns among the tissues.

**TABLE 1:** Summary statistics for splicing patterns in reciprocal crosses among tissues.

Tissue	Group	Conser vative	Additivity		CG-like dominant		WL-like dominant		Transgressivity	
			CG>WL	CG<WL	Up	Down	Up	Down	Over-dominant	Under-dominant
brain	FC	350	55	42	117	42	102	40	120	121
	MC	371	45	42	104	36	91	65	118	119
	FR	333	41	38	144	52	100	55	109	117
	MR	371	54	43	104	35	87	65	111	121
muscle	FC	808	94	84	145	121	182	117	119	128
	MC	840	103	87	111	65	250	153	91	93
	FR	769	100	68	186	222	127	86	126	130
	MR	794	91	104	160	123	195	118	100	120
liver	MC	261	29	37	86	41	46	39	78	99
	MR	259	26	28	96	47	52	33	73	90

### Functional Analysis of Non-Conserved AS Genes

**FIGURE 7:**
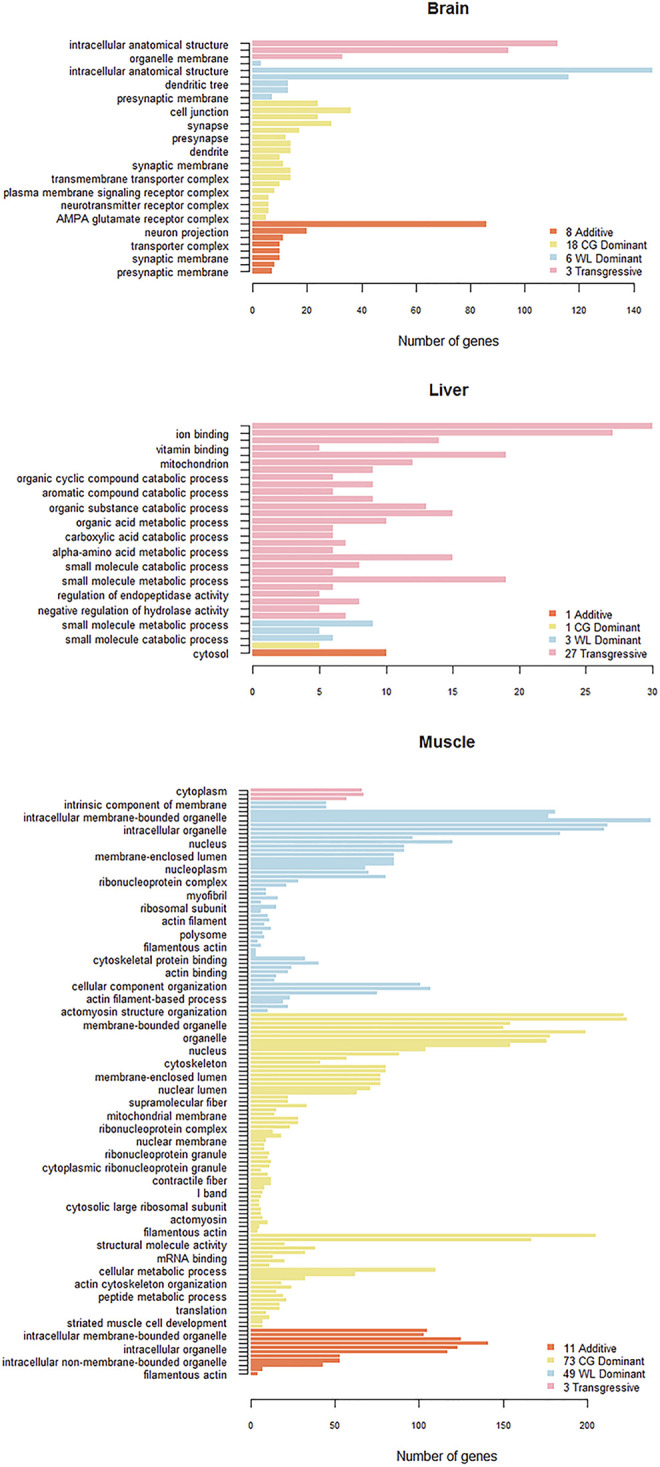
Gene Ontology (GO) analysis of un-conserved pattern AS genes in three tissues. GO functional enrichment analysis is performed on additive, dominant and transgressive AS genes in brain, liver, and muscle.

We selected non-conserved genes that showed the same pattern in a particular organ tissue in more than two groups, to ensure accuracy prior to functional annotation. There were 148, 211, 203, and 307 AS genes with additive, CG dominant, WL dominant, and transgressive patterns, respectively, in the brain tissue, while the corresponding numbers for the muscle tissue were 179, 254, 302, and 188, and those for the liver tissue were 25, 84, 46, and 125, respectively. GO functional enrichment analysis was used for of four categories (FDR < 0.05) in the brain and muscle tissues, while they were filtered for *p* < 0.001 for the liver tissue due to fewer selected gene samples (Figure 7). The results showed that expressed AS genes in the brain and muscle tissues are mostly related to cellular components such as cytoplasm and intracellular anatomical structure, which are mainly associated with biological processes including cellular amino acid catabolic process as well as small molecule metabolic and catabolic processes in the liver. Specifically, AS genes of classified non-conserved mode from the muscle tissue were involved in the development of ribosomes, and muscle structure such as actin filament, myofibril, contractile fiber, sarcomere, and sarcolemma.

**FIGURE 8:**
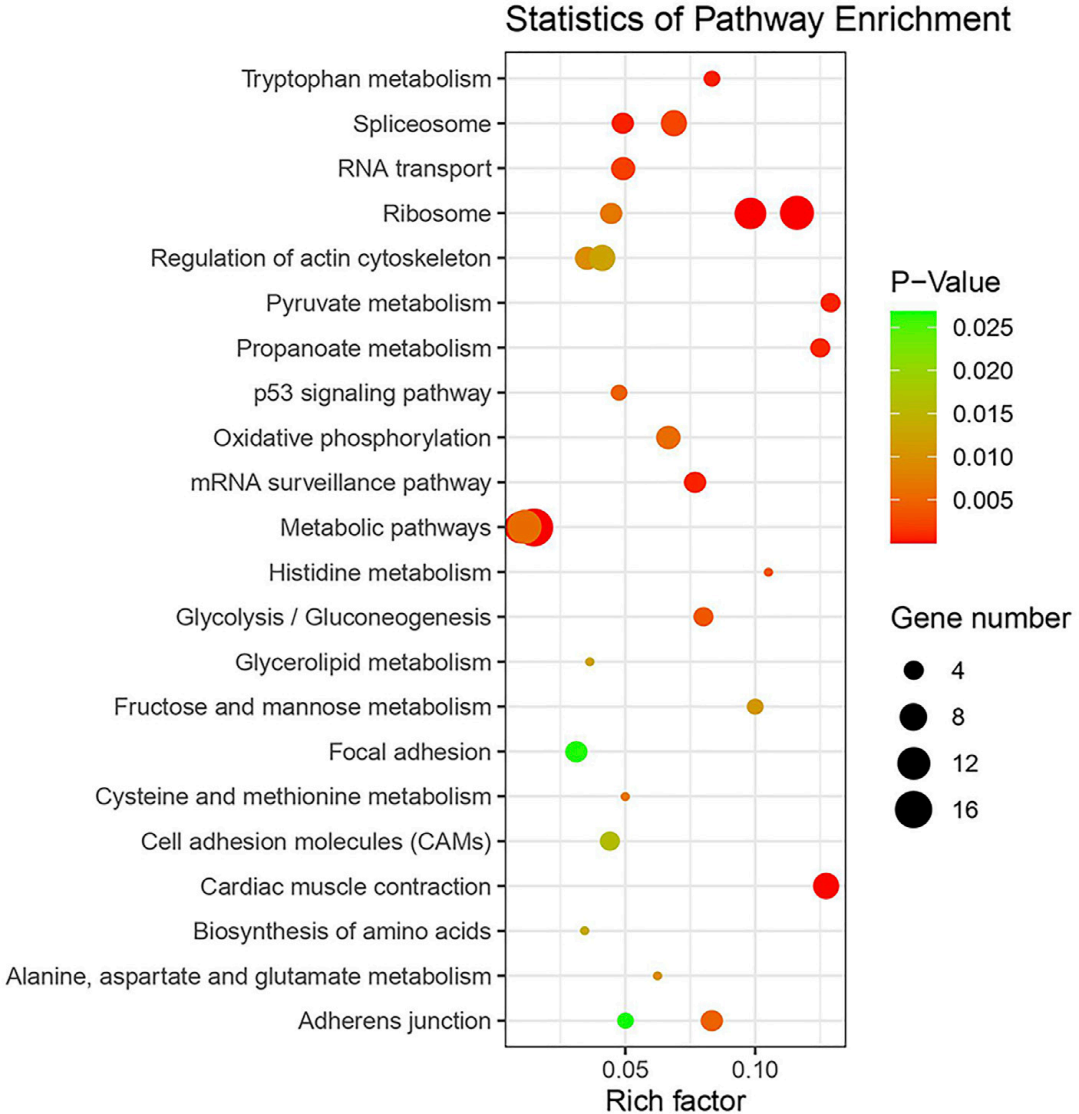
KEGG pathway enrichment analysis of AS genes in 10 most enriched pathways from each tissue.

KEGG pathway enrichment analysis was performed to identify whether AS genes are involved in signal transduction pathways and biochemical metabolic pathways. We focused on the 10 most significant pathways in each tissue (Figure 8, Supplementary Table S2). Six pathways which are involved in many important biological processes such as metabolic pathways, energy metabolism, genetic information processing in RNA translation and transcription, cellular community, and cell motility, overlap among tissues. In the brain, CG-like dominant and under-dominant genes that contained an average of 17.6 exons were involved in several pathways related to energy metabolism and RNA translation, such as mRNA surveillance, RNA transport, and ribosome and cellular processes including regulation of the actin cytoskeleton and adherens junction. Most of the CG-like dominant genes in the muscle were enriched by ribosome, spliceosome, and oxidative phosphorylation, while additive genes were associated with carbohydrate metabolism including glycolysis and pyruvate metabolism. In the liver, we found that metabolic pathways, glycolipid metabolism pathways, and several amino acid metabolism-related pathways that included tryptophan, histidine, cysteine, and methionine were significantly enriched in WL-like dominant and under-dominant genes.

## Discussion

A ubiquitous and complex component of gene regulation in humans, animals, and plants is AS(Pan et al., 2008; Barbosa-Morais et al., 2012; Marquez et al., 2012). Using comparative transcriptome analysis, a previous study had found that 23% of chicken genes undergo AS, compared to 68% in humans and 57% in mice (Elsa and Shoba, 2009). Li et al. observed that AS genes make up approximately 36.85% of a genome, and over 40% of them are specifically expressed during muscle development (Li et al., 2018). There is a growing recognition of the contributions of AS to myogenesis, and the refinement of muscle function, neuronal development, and the function of mature neurons (Bland et al., 2010; Vuong et al., 2016; Nikonova et al., 2020). On one hand, we detected over 45000 putative splicing events, where 56% of genes undergo complex splicing; SE is the most common event, while RI is the rarest, which is consistent with the findings of Rogers et al. (Rogers et al., 2021). The proportion of different splicing types is dynamic, depending on the location of tissues, species, size, and development periods. The muscle and brain showed a similar proportion of five types of events, whereas the liver exhibited slightly different results Previous studies have shown that A3SS is most common in Luning chickens and sheep, and RI is the major event in muscle tissues of the Gushi chicken (Zhang et al., 2013; Zhang et al., 2017; Li et al., 2018). Additionally, RI is the most common type of event in bovine embryos compared to calves and adults, with the frequency of all splicing events sharply decreasing after birth (Sun et al., 2015). Splicing events are unevenly distributed on chromosomes, with the frequency of events consistent with chromosome length (chromosomes-1 and chromosome-2 are the longest); a similar result was reported in a sheep study (Zhang et al., 2013). For example, in the breast muscle, more events are positioned on chromosome-7, and *MSTN* and *NEB* are specific to muscle development and growth (Donner et al., 2004; Chen P. R. et al., 2019). Most analyses are centered on the effect of spatial and temporal transcriptomes; measurement of tissue-specific differences between two modern chicken lines by us indicated that 7–11% of divergent genes (FDR < 0.05) undergo AS, with more than half the gene detected in one tissue. Compared to the muscle and liver, the brain tissue undergoes more complex splicing, and exhibits fewer divergence events than other tissues, suggesting that it is relatively conserved between broilers and layers. AS was relatively conserved in the brain tissue, with 7% fewer divergent events than in the muscle and liver tissues, indicating high tissue and strain specificity. With over 95% of splicing events being tissue-specific in both proteomics and RNA-seq analyses, the brain has strongly conserved splicing signatures, while the nervous system has also been shown to record many conserved tissue-specific splicing events (Gu et al., 2020; Rodriguez et al., 2020). Merkin et al. also indicated that splicing in the brain is well conserved, through measured transcriptome variations among multiple vertebrate species, and a similar result was also obtained by gene expression analysis (Merkin et al., 2012; Gu et al., 2020).

Approximately half of the splicing events that are significantly different between CG and WL are classified into nine types due to the divergence between hybrids and parents. Considering that the time of divergence of the two breeds is relatively recent, more than one-third of the events exhibited a conserved pattern in the three tissue types, the other patterns being the additive, dominant, and transgressive patterns. Especially in the muscle tissue, four groups consistently indicate events that are conserved during hybridization (41%); the muscle structure and basic developmental mechanisms are highly conserved (Nikonova et al., 2020). The greater prevalence of this category is also observed in coffee and Drosophila hybrids (Marie-Christine et al., 2015; Lopez-Maestre et al., 2017). We recognized that a small proportion of events were additive, while the majority of events were dominant and transgressive; similar categorization of gene expression have been reported by other studies (Gu et al., 2020). The predominance of non-additively expressed splicing in hybrids also supports the idea that most hybridizations can cause “transcriptome shock,” a widely studied phenomenon in hybrid plants, and associated with the divergence between parental species (Hegarty et al., 2006). In addition, the pattern of enhancing dominance was more prevalent than suppressing dominance in most groups, indicating that up-regulated dominant events potentially play important roles in heterosis. Previous studies on body weight in *Drosophila* and gene expression modes in the liver of chickens have shown similar results (Mai et al., 2019). However, due to different cluster methods used, other studies have reported that additivity is the main gene expression pattern in embryonic chickens (Wu et al., 2016;Zhuo et al., 2019). They classified “expression dominance” as being additive, where the genes in hybrids are not significantly different from those of one of the parents and from mid-parental values, and genes in this parent and mid-parental values are significantly different from those of the other parent (Rapp et al., 2009). This approach is more suitable for polyploid-like cotton and *cobitis* (Rapp et al., 2009;Oldřich et al., 2019). The method being unsuitable in our case, we used the Fisher test compared with the threshold criteria (Supplementary Table S1), in which more than 60% of events overlapped in each pattern. Stringent criteria may lead to slightly different results, although the two methods still draw the same conclusion.

Functional enrichment analyses in muscle tissue showed pyruvate metabolism, an intersection of key pathways of energy metabolism including *LDHB*, *LDHA*, *PDHA2*, and *ACSS2*. *LDHB* controlled lactate metabolism, and the mRNA and protein expression levels were significantly higher in wooden breast chicken (Zhao et al., 2019). *ACSS2* encodes a cytosolic enzyme that catalyzes the activation of acetate for lipid synthesis and energy generation. In addition, oxidative phosphorylation forms adenosine triphosphate (ATP) as a result of the transfer of electrons from NADH or FADH2 to O_2_ by a series of electron carriers related to six genes namely *ATP6V1A*, *UQCRQ*, *NDUFA8*, *NDUFV3*, *NDUFB3*, and *UQCRC2*. This pathway is also correlated with body weight in *Drosophila,* and is associated with heterosis in plants (Mcdaniel and Grimwood, 1971; Katara et al., 2020). By detecting ATP content and ATPase activity in reciprocal cross chickens, Mai et al. validated that oxidative phosphorylation is the major genetic and molecular factor in growth (Mai et al., 2021). The liver is the major site of amino acid metabolism and fat deposition in the body, and 37 non-conserved AS genes are significantly enriched in metabolic pathways, amino acid metabolism, and glycerolipid metabolism.

The relative frequency of cis-regulatory elements, and trans-acting BP divergence has great influence on the inheritance of gene expression patterns in hybrids (Lemos et al., 2008). Allelic-specific gene expression tests revealed that cis-regulatory divergence is a predominant contributor in mouse and Camellia, whereas trans-regulatory divergence is an important driving force in *Drosophila*, and greater parental genetic divergence decreases inheritance of patterns in coffee (Bell et al., 2013; Gao et al., 2015; Marie-Christine et al., 2015; Zhang et al., 2019). Compared with mouse and *Drosophila* inbred lines, the divergence time between commercial chicken strains is relatively short, resulting in a small number of single nucleotide polymorphisms that could be studied. In addition, short read-based data are limited to identifying allele-specific AS events, while full-length transcript sequencing might improve the accuracy and robustness of AS analysis. Studies have already explored a bulk of new transcript isoforms in chicken (Thomas et al., 2014; Sun et al., 2021); numerous studies have reported that coordinated action between AS and nonsense-mediated RNA decay controls the ratio of productive to unproductive mRNA isoforms (Garcia-Moreno and Romao, 2020). With the development of sequencing and bioinformatic analysis, further studies can explore evolution and interaction with other post-transcriptional mechanisms of AS to better understand the regulation of biological processes.

## Conclusion

In this study, AS events were observed to be highly specific to tissues and strains, and events in the brain were found to be relatively well-conserved. Meanwhile, inheritance pattern analysis of AS events showed that dominant pattern was predominant excluding conserved pattern, and indicated that a large proportion of inheritance patterns exhibited enhanced parent-specific dominance related to heterosis in chickens. These findings provide new insights into the genetic mechanisms underlying hybridization.

## Data Availability

The original contributions presented in the study are publicly available in NCBI under accession number PRJNA591354.
