# Supplementary figures and images for: Transcriptome-Wide Analyses Identify Dominant as the Predominantly Non-Conservative Alternative Splicing Inheritance Patterns in F1 Chickens
Source: Front Genet. 2021 Dec 3;12:774240. doi: 10.3389/fgene.2021.774240 (PMC8678468; doi:10.3389/fgene.2021.774240)

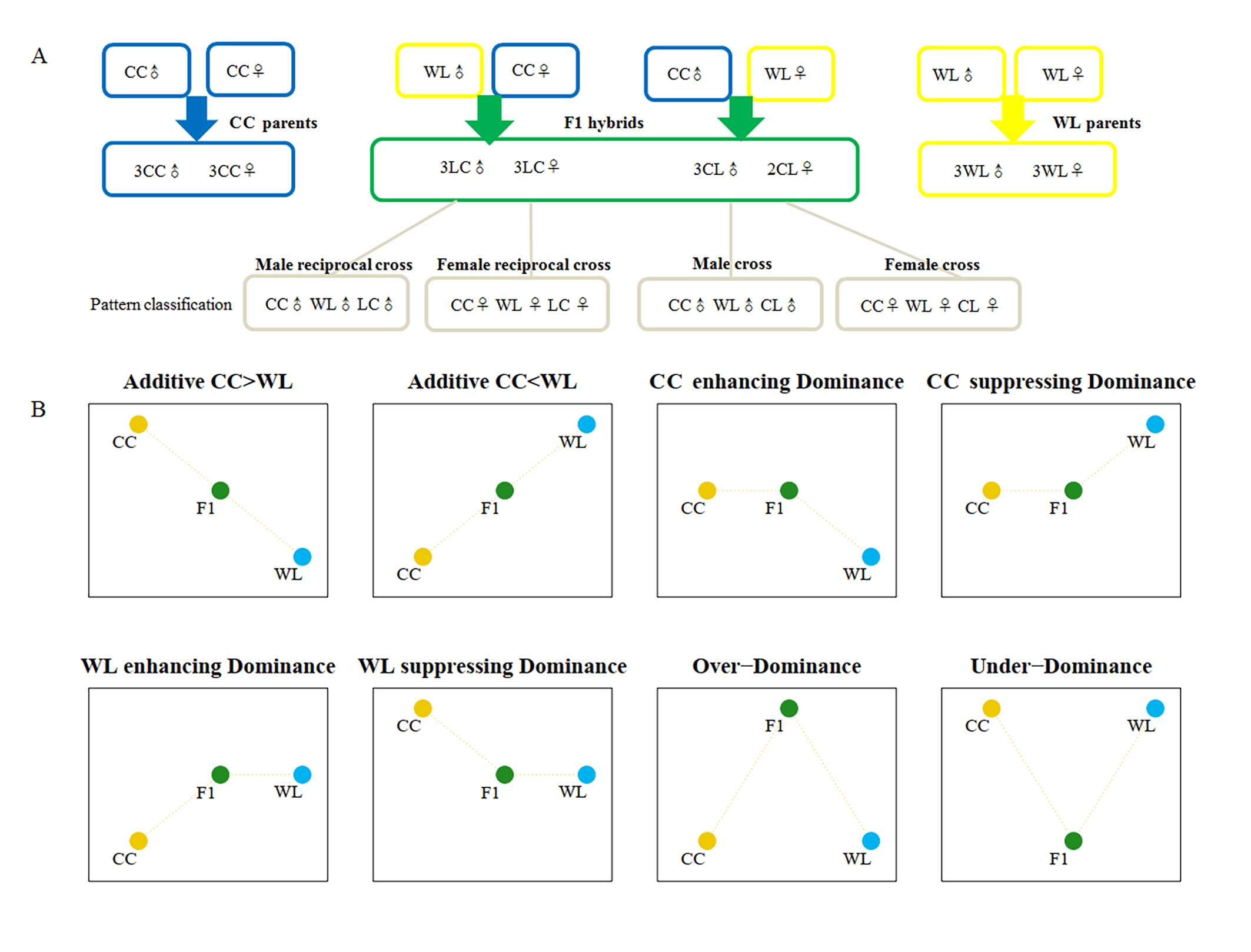

Supplement: Supplementary file 2 [file Image1.TIF]
